# Supplementary material for: Chromothripsis during telomere crisis is independent of NHEJ, and consistent with a replicative origin
Source: Genome Res. 2019 May;29(5):737–49. doi: 10.1101/gr.240705.118 (PMC6499312; doi:10.1101/gr.240705.118)
Supplement: Supplemental Material [file supp_gr.240705.118_Supplemental_file_1.zip › contigs/annotated_contigs/DB55/contig.2.DB55_length_554_mean_cov_10.5920577617.docx]

**DB55_length_554_mean_cov_10.5920577617**

```
ACAATTTACAGTTTACACATTTATGAACCCAGAGGAAATGCTCCAGCATACGGAATGTACAATTGTTACCGTTTACATTTAAATTTTTC
 >chr7:147187218-147187513 - E=7e-166
TCTAACATTGGCTAGAAAAAATTTTACTTTTGCTTTATTTGGCAATGTCAGGACCTGTTCCTTACTAGTATTTCAGAATTTTTAAATCT

CGTGATTTTAAAATTAAAAGAGAATTATATAGGTCATCTAGTTTATTCCTCCTGTTTCCCCTGGAAACATTACGTCTATATATTTGGGC

AACATGAAAATCTCTCCTTTAATAAAAA|AGCACTTTGGAAGGCCGAGGCGGGCGGATCACCTGAGGTCAGGAGTTCGAGACCAGCCTG
                              >chr7:146900332-146900591 - E=3e-138
GCCAACATGGTGAAACACTATCTCTACTAAAAATACAAAAATTAGCTGGGCATGGTGGTGGGTACCTGTAATCCCAGCTACTGGGGAGA

ATTGCTTGAACCTGGGAGGCCGAGTTTGCAGTGAGCCAAGATCACACCATTGCACTCCAGCCAGGGCAACAGAGTGAGACTCCATCCCA

AAATAAATTAAAATATTAAAT
```
